# Whole-exome sequencing identifies a novel mutation in spermine synthase gene (SMS) associated with Snyder-Robinson Syndrome

**DOI:** 10.1186/s12881-020-01095-x

**Published:** 2020-08-24

**Authors:** Talal J. Qazi, Qiao Wu, Ailikemu Aierken, Daru Lu, Ihtisham Bukhari, Hafiz M. J. Hussain, Jingmin Yang, Asif Mir, Hong Qing

**Affiliations:** 1 Key Laboratory of Molecular Medicine and Biotherapy, Department of Biology, School of Life Science, Beijing Institute of Technology, Beijing, China; 2 State Key Laboratory of Genetic Engineering, School of Life Sciences, Fudan University, Shanghai, China; 3 Chongqing Population and Family Planning, Science and Technology Research Institute, National Health and Family Planning Commission, Chongqing, China; 4 Key Laboratory of Helicobacter pylori and Microbiota and GI Cancer in Henan Province, Marshall Medical Research Center of Zhengzhou University, The 5th affiliated Hospital of Zhengzhou University, Zhengzhou, China; 5 Department of Nephrology, Institute of Nephrology, Shanghai Ruijin Hospital, Shanghai Jiao Tong University, School of Medicine, Shanghai, China; 6 Shanghai WeHealth Biomedical Technology Co., Ltd., Shanghai, China; 7 Department of Biological Sciences, FBAS, International Islamic University, Islamabad, Pakistan

**Keywords:** Snyder-Robinson syndrome, *SMS*, X-linked mental retardation, Intellectual disability, Gait abnormalities

## Abstract

**Background:**

Loss of function mutations in the spermine synthase gene (SMS) have been reported to cause a rare X-linked intellectual disability known as Snyder-Robinson Syndrome (SRS). Besides intellectual disability, SRS is also characterized by reduced bone density, osteoporosis and facial dysmorphism. SRS phenotypes evolve with age from childhood to adulthood.

**Methods:**

Whole exome sequencing was performed to know the causative gene/pathogenic variant. Later we confirmed the pathogenic variant through Sanger sequencing. Furthermore, we also performed the mutational analysis through HOPE SERVER and SWISS-MODEL. Also, radiographs were also obtained for affected individual to confirm the disease features.

**Results:**

In this article, we report the first Pakistani family consisting of three patients with SRS and a novel missense pathogenic variant in the *SMS* gene (c.905 C > T p.(Ser302Leu)). In addition to the typical phenotypes, one patient presented with early-onset seizures. Clinical features, genetic and in-silico analysis linked the affected patients of the family with Snyder-Robinson and suggest that this novel mutation affects the spermine synthase activity.

**Conclusion:**

A novel missense variant in the SMS, c.905C > T p. (Ser302Leu), causing Snyder- Robinson Syndrome (SRS) is reported in three members of Pakistani Family.

## Background

Polyamines are organic compounds having more than two amino groups. At neutral pH, they exist as ammonium derivatives. These are polycations that can interact with negatively charged particles, i.e. DNA, RNA and some negatively charged proteins.

Polyamines play an essential role in cell growth, survival and proliferation. In addition to this, half of the polyamines results from the activity of spermidine synthase to convert putrescine into spermidine and spermine synthase to convert spermidine into spermine [1–3].

Snyder-Robinson syndrome is a rare disorder with an unknown prevalence [4]. Worldwide, around 10 families, segregating this disorder in 20 patients with 11 mutations, have been identified so far. Other names for this disorder include: X-linked syndromic mental retardation, Snyder-Robinson type; Snyder-Robinson X-linked mental retardation syndrome and spermine synthase deficiency (Genetics Home Reference) [4]. Snyder-Robinson syndrome (OMIM #309583, SRS) is caused by loss of function mutations in the spermine synthase gene (OMIM #300015, SMS). This is an X-linked disorder first time identified in 1969 [5]. The phenotype was better defined in a re-evaluation of the original family, and linkage analysis localized the related gene to Xp21.3–p22.12 [6].

The life of patients with SRS (OMIM #309583) has several burdens, not limited to osteoporosis. Intellectual disability, seizures, kyphosis and scoliosis are additional manifestations that cause disability in these people. In the affected individuals, *SMS* hemizygous pathogenic variant results in reduced activity of *SMS* activity and decreased spermine-spermidine ratio [7]. The daily life routine, of the individuals suffering from SRS, is significantly disturbed, also having atraumatic osteoporotic features in addition to above symptoms. Osteoporosis is a disease in which density and quality of the bone are reduced and it also termed as Porous Bones. This arises from the disruption of the equilibrium between osteoclastic bone reabsorption and osteoblastic bone.

In the present research, we report the investigations of a family (SRS1) from Pakistan, segregating SRS in a pattern consistent with X-linked recessive inheritance. We report a missense pathogenic variant in this family and it is the first case reported from Asia.

## Methods

### Family recruitment and neurodevelopmental assessment

**Fig. 1:**
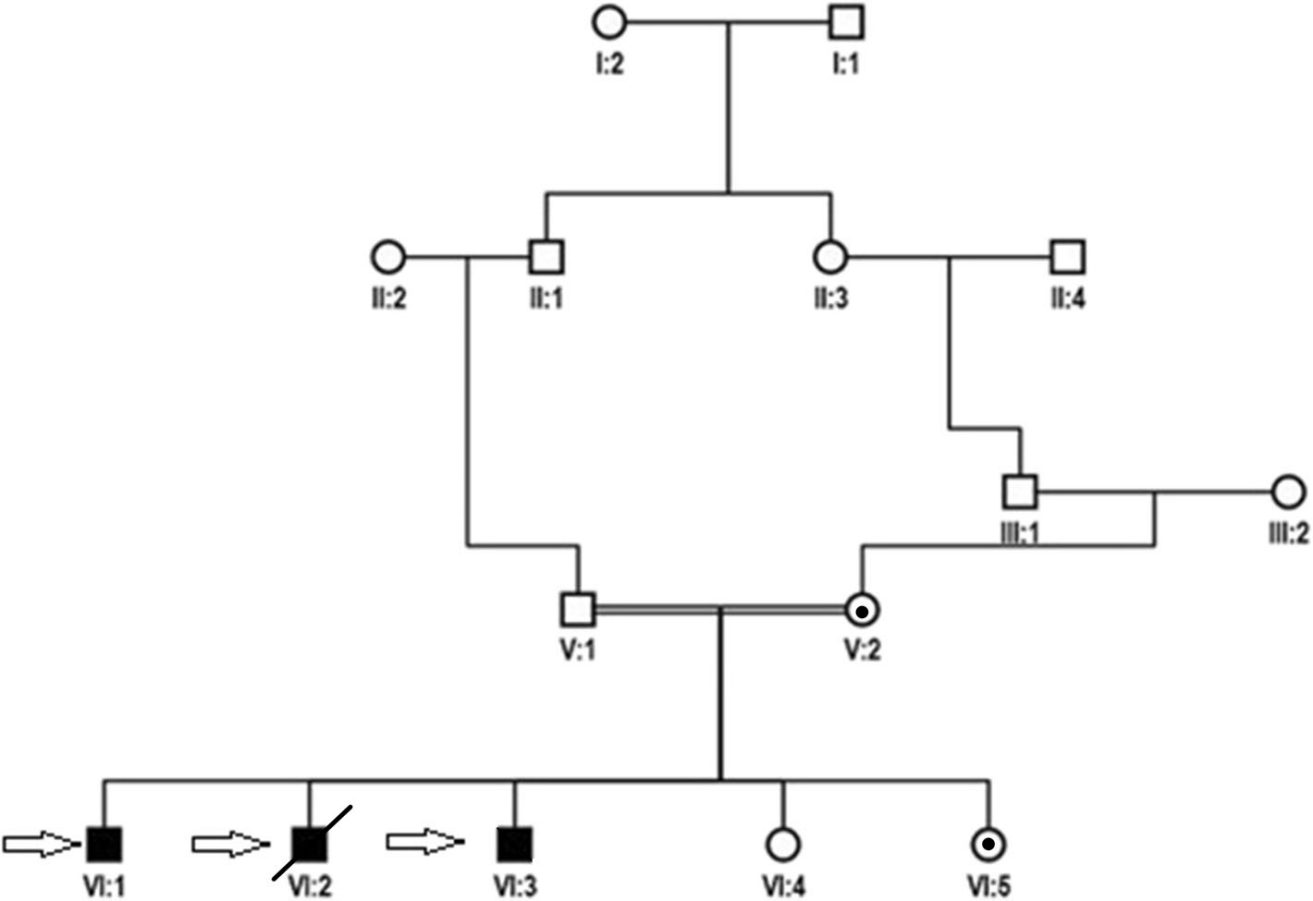
Family pedigree and Sanger sequencing confirmation of the novel c.905C > T SNV variant. Black symbols represent affected individuals. The index patients are indicated with an arrow. Dot inside the circle indicates carriers

A family from Vehari District, Punjab Province, Pakistan, after informed written consent was recruited at International Islamic University, Islamabad (IIUI). IIUI also approved this study under the protocol No. IIU (BI & BT) FBAS-2017. The family has three affected individuals in the same generation from parents with consanguineous relationships **(**Fig. 1**)**. In order to evaluate the intellectual disability degree of affected members and their psychological and neurological assessments were conducted by experienced doctors at the Alkhidmat Raazi Hospital, Islamabad. Also, a psychiatrist trained and experienced in intellectual disability psychiatry, which also came from the same cultural and lingual background as of the family, evaluated the patients using the Vineland Adaptive Behavior Scales, Second Edition [8].

### Whole-exome sequencing and data analysis

The process of whole-exome sequencing was done in Shanghai WeHealth Biomedical Technology Co., Ltd. All genomic DNA samples from patients and their family members were extracted from peripheral leukocytes using a commercial kit (TIANGEN, China). The quantity/quality of DNA was analyzed by NanoDrop ND-1000 (Thermo, USA) spectrophotometer and by agarose gel electrophoresis. Exome capture was performed with xGen Exome Research Panel v1.0 (IDT, USA) and 150 base pair paired-end sequencing was executed using the Illumina HiSeq platform (Illumina, USA). The raw reads were aligned by the sequencing company using the Burrows-Wheeler Aligner (BWA) and SAM tools. Then after removing duplicates from the sorted alignment using Picard, variants were called using the Genome Analysis Toolkit (GATK v3.70) pipeline. Variants were classified according to the American College of Medical Genetics guidelines [9].

### Variant confirmation

Primer3Plus browser was used to design the oligonucleotides, flanking the genomic location of the identified variant. Polymerase chain reaction (PCR) amplifications were performed using genomic DNA of the proband and accessible family members for the confirmation of the veracity of the likely causative variant and to assess segregation within the family. PCR reactions were performed in 20 ul volumes (2xTaq plus Master Mix, P211-AA) with the following primers: 5′-GCAGTGCTAGGTGGATGTGATT-3′ and 5′-AATCCGATGATGCCGCTCTATC-3′, with an annealing temperature of 58 *°*C. PCR products were, unidirectional, sequenced using Big Dye Terminator v3.1 on ABI 3730XL sequencer (Applied Biosystems/Life Technologies, Carlsbad, CA). Sequences were manually reviewed and compared to reference sequence NM 004595.4 of SMS gene using Codon Code Aligner software.

### Mutation analysis

‘The structural information of human wild type Spermine Synthase was obtained from Protein Data Bank (PDB ID: 3C6K) [10, 11]. Annotations about this protein were obtained from UniProtKB entry P52788. HOPE SERVER was accessed to analyze the results [12]. Besides, 3D protein structure model was built by using SWISS-MODEL. Wincoot software was used for introducing pathogenic variants to structure, and Software PyMOL software was used to represent structural figures [13, 14].

## Results

### Clinical details

#### Patient (VI: 1)

The Proband (VI: 1) is 18-years-old boy born to healthy parents and family history was unremarkable. His birth weight and occipitofrontal circumference (OFC) were 2.20 kg and 34 cm, respectively. He cannot stand and walk, only move by crawling. He has global developmental delay. He has bulging (pectus carinatum) with no other facial dysmorphic features. The patient exhibited severe dysarthria but did not complain about any visual and auditory problems (Table 1).

**Table 1:** Clinical representation of affected individuals in family

Clinical features	Patient 1 (VI:1)	Patient 2 (VI:2)	Patient 3 (VI:3)
Age	18	10	8
Intellectual disability	+ (mild)	+ (mild)	+ (mild)
Bone abnormality	+	+	+
Prominent lower lip	–	+	+
Speech abnormalities	Echolalia	Slow	Slow
marfanoid habitus	–	–	–
Ambulatory difficulties	limited	limited	limited
Low muscle mass	+	–	–
Kyphscoliosis	+	–	–
High narrow or cleft palate	+	+	+
Facial asymmetry	–	–	–
Unsteady gait	–	–	–
Long toes	+	–	–
hypotonia	–	–	–
Nonspecific movement disorder	–	–	–
Seizures	+	+	+
Long hands with large fingers	+	–	–

#### Patient (VI: 2)

This patient (VI: 2), second of three affected siblings, unfortunately died during the study. By the time of his death, he was 10-year-old. He was born after an uneventful pregnancy and his weight and OFC were 2.27 kg and 37 cm, respectively at birth. He had facial dysmorphic features including a long oval, midface hypoplasia. He had been suffering from respiratory secretions. He had frequent seizures, hypotonia, decreased muscle bulk, and flexion contraction of the large and small joints. He was not able to stand independently and could only move by crawling. He had skeletal problems, including bone fractures of his distal fibula and spine problem. An EEG of the patient manifested slowing background at 14 months of age with no other abnormalities (Table 1).

#### Patient (VI: 3)

The patient (VI:3) is the 8-year-old boy with the complaint of severe pain in bones, hypotonia, regression and lost motor skill in the first 2 years of life. An EEG at 14 months of age showed generalized slowing and later on, manifested seizures. He had walking problems at an early age. He has multiple traumatic fractures in tibia, femur and humerus (Table 1).

### Genetic analysis

**Fig. 2:**
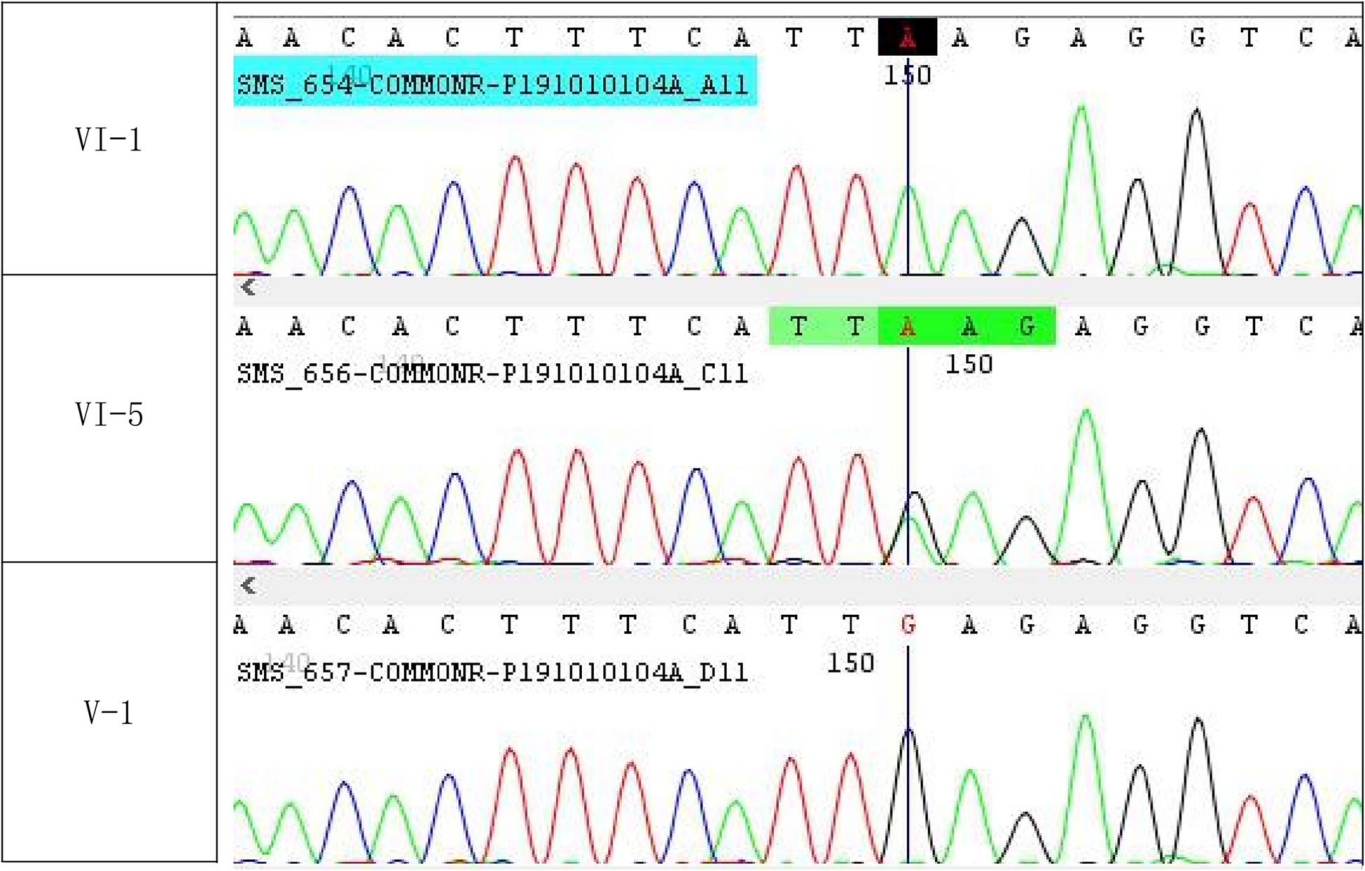
Sequence chromatograms of the region including the variation c.905C > T in SMS gene of a normal individual (V:1), an obligate carrier (VI:5) and an affected individual (VI:1). A straight line indicates the position of variation on chromatogram

The variant NC_000023.10 g. 22003301C > T; NM_004595.4 c.905C > T p. (Ser302Leu) was identified in the SMS gene in the index patient (V: 1), through whole-exome sequencing analysis. This variant was then confirmed by Sanger sequencing in his brother (VI: 2) and revealed that their mother and sister (V: 2; VI: 5) are heterozygous; the father (V: 1) and grandmother (paternal side) (II: 2) are normal (Fig. 1). Wild type, hemizygous and heterozygous electropherograms are shown in Fig. 2. The pathogenic variant was absent in the general population (gnomAD https://gnomad.broadinstitute.org/). These results indicate that this rare SNV co-segregates with the patients’ phenotypes. Since only male carriers showed disease phenotype, the inheritance pattern of this disease matches XLR. Genotypes and Sanger Sequencing of the family members who participated in study is given in Supplementary file.

### Effect of mutation on protein

**Fig. 3:**
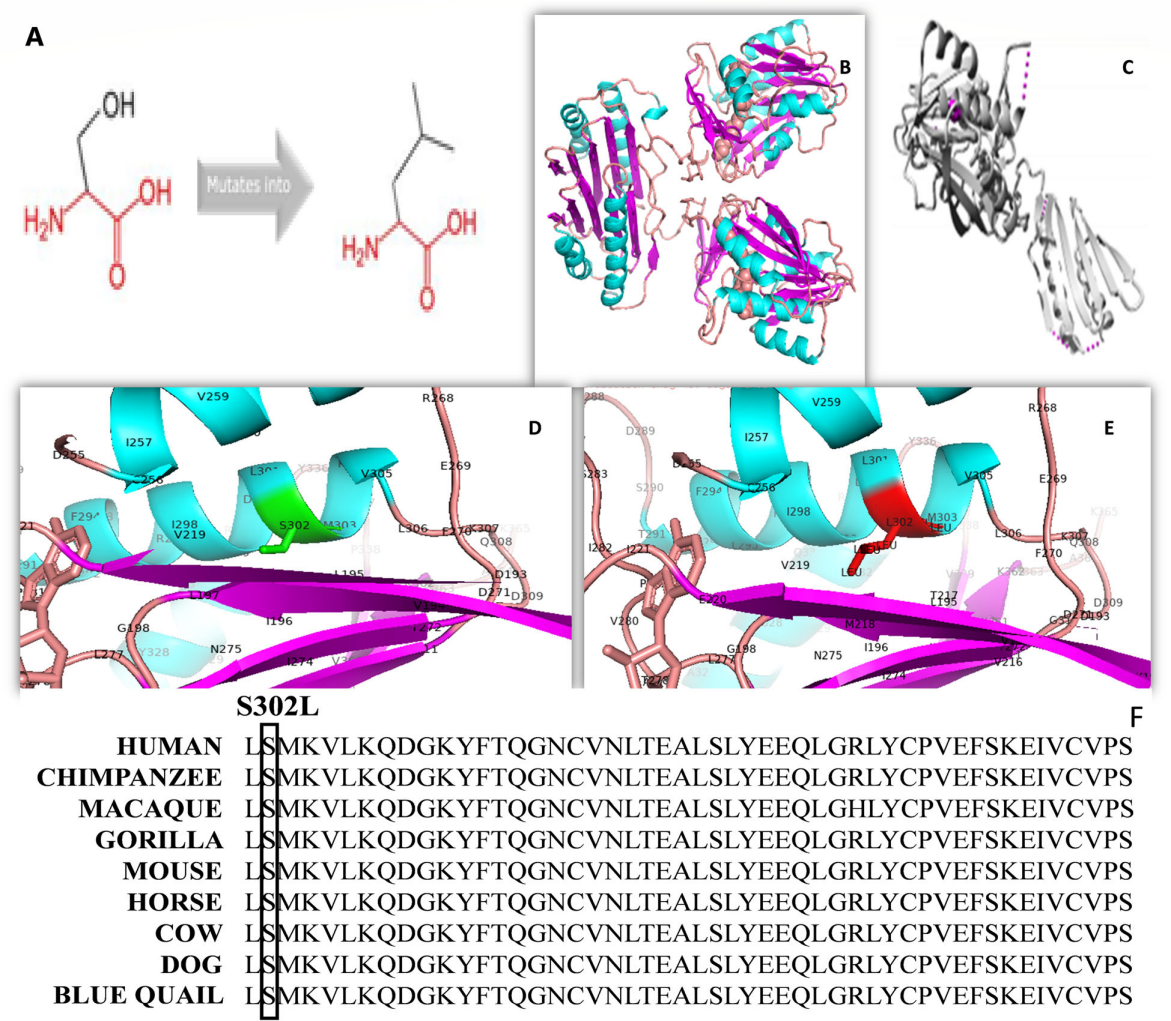
**a** Structural formulas show that the mutant amino acid residue is bigger than the wild-type amino acid residue (**b**) 3D structure of wild-type of Human *SMS* protein in ribbon presentation. Helices (shown in cyan color), Sheets (shown in magenta color) and loops (shown in orange color). **c** Overview of the protein in ribbon-presentation. The protein is colored grey, the side chain of the mutated residue is colored magenta and shown as small balls. **d** Zoomed 3D structure of wild-type of human *SMS* in ribbon presentation Helices (shown in cyan color), Sheets (shown in magenta color) and loops (shown in orange color). Serine is present at position 302 shown in green color. **e** Zoomed 3D structure of mutant *SMS* of human in ribbon presentation Helices (shown in cyan color), Sheets (shown in magenta color) and loops (shown in orange color). Serine is replaced by Leucine at position 302 shown in red color (**f**) Sequence alignment of SMS gene among different species. In the human sequence, amino acids from 301 to 360 are shown. The mutation site considered in this study was showing complete conservation among different species. Multiple sequence alignment is performed with Clustal Omega protein alignment tool

The 3D-structure of our protein of interest was already available. To investigate pathogenic variant effect on protein, schematic structures of the WT (left) and the mutant (right) amino acids are shown in Fig. 3.

Pathogenicity resulting from missense mutation would derive from a misfolding generated substantially by three factors: a) different steric hindrance of the residue (the new residue has a larger size), b) different hydrophobicity, which also prevents the new amino acid from creating a hydrogen bond with Ile in position 298 (and the hydrogen bond network is important for the enzymatic functionality) c) position within the protein core where there is no space to accommodate larger residues. Report can be retrieved from link: https://www3.cmbi.umcn.nl/hope/report/5f089c8bfc0fd33a55c7246d/.

## Discussion

**Fig. 4:**
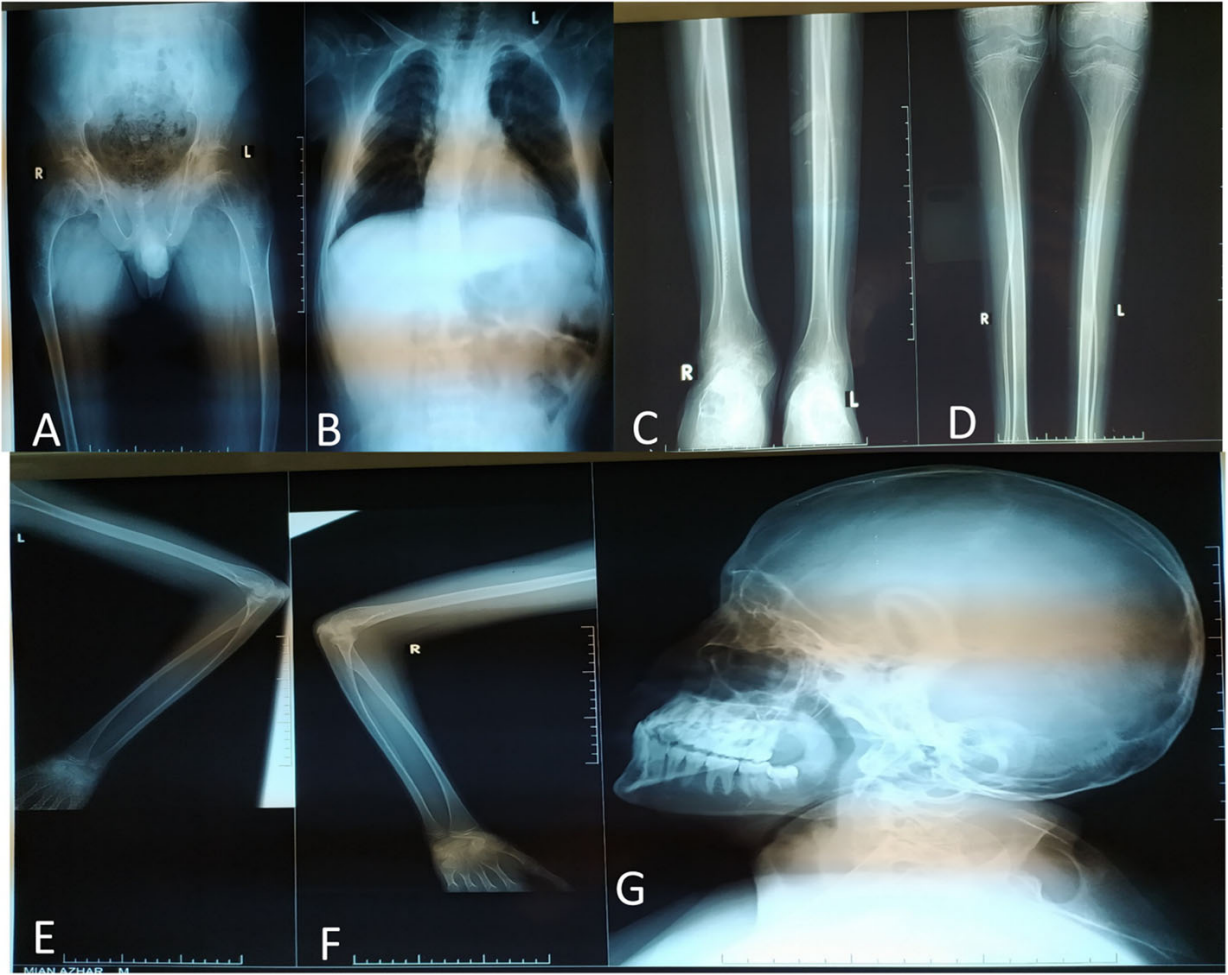
Radiographic findings in Patient VI:3. **a**. Frontal radiograph of the pelvis shows increased bone density, trabecular thickening and ossification of the sacrotuberous ligament. Mild flattening of the acetabular roof is noted giving rise to champagne glass deformity of the pelvis. **b** Both lung fields are clear Bilateral CP angles are sharp. Mediastinal contours appear unremarkable. Scoliotic deformity of the spine is noted with convexity towards right side. **c** Outward bowing of bilateral femur is noted. **d** Bilateral fibula shows marked thinning and outward bowing (**e**, **f**) outward bowing of bilateral humerus bone is also noted. **g** Long oval face hypoplasia and bone deformities

Till date, 11 hemizygous variants in *SMS* gene causing Snyder-Robinson syndrome so far reported [6, 15, 16]. All of these were missense mutations and a case of complete LoF variant in SMS is reported in 2020 [17]. Here, we identify the first Pakistani family with a novel pathogenic variant in the *SMS* gene, which expands the phenotypes and focuses on the characteristics of SRS. The pathogenic variant was absent in the general population (gnomAD https://gnomad.broadinstitute.org/). In this family, patients presented all the clinical features previously described in SRS [6], such as ID, facial dysmorphic features, including long oval midface hypoplasia and bone deformities (Fig. 4). There is wide phenotypic variability in the reported SRS patients, however, as yet, no genotype-phenotype correlation has been described. The SMS gene codes for an enzyme called spermine synthase whose function is the production of spermine from spermidine for polyamine metabolism.

The reported pathogenic variant p.S302L is the substitution of amino acid residue serine by a residue leucine. The pathogenic variant site S302 is buried in the protein interior. As it is shown in Fig. 3d and f, the structure around the S302 pathogenic variant site is present in a very packed and conserved area, and there is no space to adjust the amino acid leucine. The mutated residue is located in a domain that is important for the activity of the protein and in contact with another domain that is also important for the activity. The interaction between these domains could be disturbed by the mutation, which might affect the function of the protein. This pathogenic variant could reduce the level of spermine synthase in the body with increased spermidine/spermine ratio causing the disorder in affected Individuals.

The overall study revealed the molecular mechanism of the causative mutation in SMS gene. As most of the SUMOylation sites follow a canonical consensus motif of ψ -K-X-E/D (ψ, a hydrophobic amino acid, such as A, I, L, M, P, F, V or W; X, any amino acid residue) and this protein has the motif (ψ -K- X-E/D), it may be the target of SUMOylation. The amino acid position 302 is located very close to this motif (297-LILDLS/LMKVLKQD-309, where the S of S/L is WT type, L is the mutant and the bold is the SUMOylated candidate motif) [18, 19], suggesting that the mutation could lead to an alteration of the putative SUMOylation process. Moreover, the pathogenic variant site is highly conserved among the species (Fig. 3f). Therefore, this mutation could result in its failure of post-translational modification by SUMOylation, with consequences on its stability. As the structural integrity of the protein is challenged, this could lead to its degradation. The degradation of Spermin Sintase protein causes the X-linked recessive Snyder-Robinson Syndrome.

## Conclusion

In conclusion, only few pathogenic variants have been reported to Snyder-Robinson syndrome. We identify the first Pakistani family carrying a novel variant in the SMS gene, contributing to broad the phenotype associated to this rare syndrome.

## Supplementary information

**Additional file 1.**

## Data Availability

WES analysis reported submitted in HARVARD dataverse, available online by using following link: 10.7910/DVN/9NP4JV and WES excel file available online by using following link: 10.7910/DVN/ELJ2ZM. The mutation identified in this study is deposited in the ClinVar repository and available online by using accession number: SCV001370544. Protein sequence of the SMS gene obtained from the UniPortKB with accession number: P52788. However additional information is provided in supplementary file. The 3D protein structure model was built by using SWISS-MODEL. Winccot software was used for introducing mutations to structure and Structural effects of a mutation are analyzed through HOPE server. PyMOL was used for representing structural figures. Report can be retrieved from link: https://www3.cmbi.umcn.nl/hope/report/5f089c8bfc0fd33a55c7246d/.

## Abbreviations

SRS: Snyder-Robinson Syndrome
ID: Intellectual Disability
WES: Whole Exome Sequencing
SMS: Spermine Synthase
WT: Wild Type
PDB: Protein Data Bank

## References

[1] Pegg AE (2009). Mammalian polyamine metabolism and function. IUBMB Life.

[2] Thomas T, Thomas TJ (2001). Polyamines in cell growth and cell death: molecular mechanisms and therapeutic applications. Cell Mol Life Sci.

[3] Kusano T, Berberich T, Tateda C, Takahashi Y (2008). Polyamines: essential factors for growth and survival. Planta..

[4] Snyder-Robinson syndrome - Genetics Home Reference - NIH. https://ghr.nlm.nih.gov/condition/snyder-robinson-syndrome#statistics. Accessed 10 Jan 2020.

[5] Snyder RD, Robinson A (1969). Recessive sex-linked mental retardation in the absence of other recognizable abnormalities: report of a family. Clin Pediatr (Phila).

[6] Arena JF, Schwartz C, Ouzts L, Stevenson R, Miller M, Garza J (1996). X-linked mental retardation with thin habitus, osteoporosis, and kyphoscoliosis: linkage to Xp21.3-p22.12. Am J Med Genet.

[7] de Alencastro G, McCloskey DE, Kliemann SE, Maranduba CMC, Pegg AE, Wang X (2008). New SMS mutation leads to a striking reduction in spermine synthase protein function and a severe form of Snyder-Robinson X-linked recessive mental retardation syndrome. J Med Genet.

[8] Sparrow SS (2011). Vineland adaptive behavior scales. Encyclopedia of clinical neuropsychology.

[9] Richards S, Aziz N, Bale S (2015). Standards and guidelines for the interpretation of sequence variants: a joint consensus recommendation of the American College of Medical Genetics and Genomics and the Association for Molecular Pathology. Genet Med.

[10] RCSB PDB - 3C6K: Crystal structure of human spermine synthase in complex with spermidine and 5-methylthioadenosine. https://www.rcsb.org/structure/3C6K. Accessed 19 Jan 2020.

[11] Wu H, Min J, Zeng H, McCloskey DE, Ikeguchi Y, Loppnau P (2008). Crystal structure of human spermine synthase: implications of substrate binding and catalytic mechanism. JBiolChem..

[12] Venselaar H, te Beek TAH, Kuipers RKP, Hekkelman ML, Vriend G (2010). Protein structure analysis of mutations causing inheritable diseases. An e-science approach with life scientist friendly interfaces. BMC Bioinformatics.

[13] Emsley P, Lohkamp B, Scott WG, Cowtan K (2010). Features and development of coot. Acta Crystallogr Sect D Biol Crystallogr.

[14] DeLano WL. The PyMOL Molecular Graphics System. San Carlos: DeLano Scientific; 2002.

[15] Becerra-Solano LE, Butler J, Castañeda-Cisneros G, McCloskey DE, Wang X, Pegg AE (2009). A missense mutation, p.V132G, in the X-linked spermine synthase gene (SMS) causes Snyder-Robinson syndrome. Am J Med Genet Part A..

[16] Peron A, Spaccini L, Norris J, Bova SM, Selicorni A, Weber G (2013). Snyder-Robinson syndrome: a novel nonsense mutation in spermine synthase and expansion of the phenotype. Am J Med Genet Part A.

[17] Larchera L, Norrisb JW, Burattia ELJ, Mignotac C, Gareld C, Kerena B, Schwartzb CE, Whalenc S (2020). The complete loss of function of the SMS gene results in a severe form of Snyder-Robinson syndrome. Eur J Med Genet.

[18] GeneCards (2018). Zfhx4. LIPA GENE.

[19] Hendriks IA, Souza RCJD, Yang B, Vries MV, Vertegaal ACO (2015). Uncovering global SUMOylation signaling networks in a site-specific manner. Nat Struct Mol Biol.

