# Supplementary figures and images for: Whole-exome sequencing identifies a novel mutation in spermine synthase gene (SMS) associated with Snyder-Robinson Syndrome
Source: BMC Med Genet. 2020 Aug 24;21:168. doi: 10.1186/s12881-020-01095-x (PMC7446199; doi:10.1186/s12881-020-01095-x)

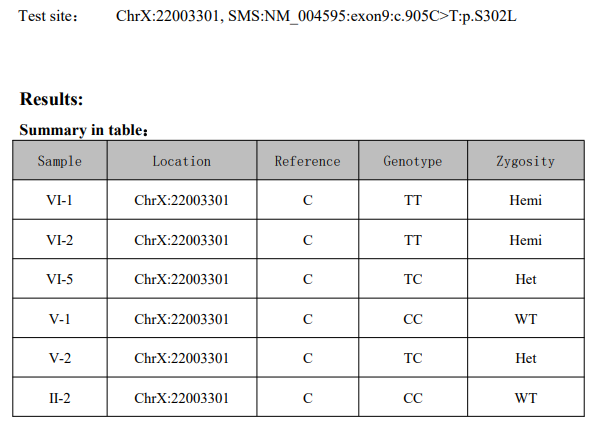


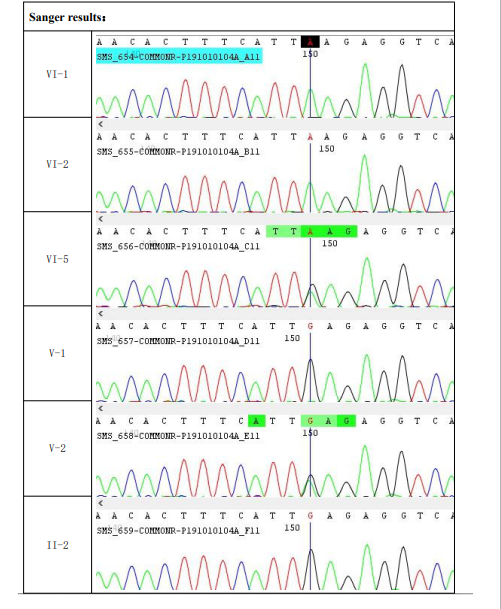

Supplement: Supplementary file 1 — Additional file 1. [file 12881_2020_1095_MOESM1_ESM.docx]
